# Railway Corporations and Their Duties to Persons Injured by Accidents

**Published:** 1888-11

**Authors:** R. M. Swearingen

**Affiliations:** Austin, Texas


DANIEL'S

### Texas Medical Journal.

A Representative Organ of the Medical Profession, and an Exponent of Rationa Medicine; devoted to the Organization, Advancement and Elevation of the Profession in Texas.

Published Monthly.—Subscription $2.00 a Year.

Vol. IV. AUSTIN, NOVEMBER, 1888. No. 5.

### Original Articles.

CONTRIBUTED EXCLUSIVELY TO THIS JOURNAL.

*The Articles in this Department are accepted and published with the understanding that we are not responsible for, nor do we indorse the views and opinions of the writers by so doing.*

### RAILWAY CORPORATIONS AND THEIR DUTIES TO PERSONS INJURED BY ACCIDENTS.

By R. M. Swearingen, M. D., Austin, Texas.

[Read at the meeting of the Austin District Medical Society, and by unanimous vote of the Society ordered to be published in the Society's official organ, Daniel's Texas Medical Journal.]

THE MEMBERS of a railway corporation may be charitably disposed, and in the private walks of life may perform many generous deeds; but when acting in the body corporate they attend strictly to financial affairs, without apparently considering the moral obligations that must inevitably fall upon them.

Their superintendents are first-class men, with splendid business qualifications,—but from the force of circumstances they acquire the habit of looking at all things from a standpoint of profit and loss.

These corporations employ the most skillful lawyers, and expect them, in every professional act, to reflect, not the will of a man, or men, but the will of a corporation.

The old fashioned scales of equity used in the ox-cart days for balancing rights and wrongs, are thrown aside by these rigid interpreters of law, and the voice of mercy seems lost in the rattle of wheels, and the scream of engines.

It is the veriest folly for any one to bring suit against a railway corporation, unless his claim is as unanswerable as a promissory note, and the law applicable to it as explicit as the ten commandments. Those who have indulged in the luxury of a lawsuit without these essential prerequisites, are generally satisfied with the one experiment.

Baffled in securing their rights, taxed by the courts for the valuable information given in the school of experience—and by their own lawyers for every note chanted in the gamut of justice, it is not surprising that these victims of lawsuits, and their friends, should look *outside* of *court-honses* for ways and means of redress, and that the strategy of war suggested the combination of forces.

All the employes of these giant corporations, believing the law inadequate to their protection, have organized into secret aid societies, in order to protect each other. Engineers, conductors, brakemen and Knights of Labor are linked together for reciprocal safety, while the Farmers' Alliance looms up in the background—a kind of reserve battle ground for all of these combinations to rally upon.

That unnamed element of discord between poverty and wealth, between labor and capital, has never before succeeded in having the lines so sharply drawn, and so clearly defined as now, and they present subjects worthy of the profoundest thought of every patriotic citizen.

Our State legislatures have been flooded with bills to regulate these colossal powers,—some of them so damaging to railway interests as to virtually amount to confiscation,—and they were, and always ought to be, defeated. Other bills were more temperate and just, but in trying to embrace the whole subject—to formulate, as it were, a great rule by which to solve many complex problems—they signally failed to accomplish anything.

The lessons taught by these failures should incline the legislative mind to easier and less vulnerable methods of reform. Whenever an evil is made apparent, correct it by a special enactment, and thus by slow, but progressive steps, remedies can be more successfully applied. Inspired by the hope of promoting such a policy, your attention is respectfully invited to the duties and responsibilities of railway corporations to persons injured by accidents.

The medical departments of these corporations are organized exclusively for the benefit of employes. The *hospital fund* defrays all expenses, and it is made from a monthly tax of fifty cents levied upon every one employed by the company. The system is a masterly creation! a kind of joint stock association, where the stockholders have no vote in the selection of officers, no voice as to the manner of investments, and no knowledge of the amounts disbursed. It is, however, an economic institution, and when properly administered furnishes comfortable quarters and skillful surgeons for both sick and wounded.

As might be expected, in this, as well as in all other extensive enterprises, incompetent men will secure positions, and either abuse or neglect the duties confided to them. For instance, the superintendent, or it may be, some official of the medical department, in their efforts to practice a questionable economy, very often rush injured men to hospitals, who are not in a condition to travel.

I can recall several such cases, and in order to more fully elaborate the position taken, will report one, more aggravated than any of the others, but no more illustrative of the evil complained of.

About five years ago, a section hand at Manor was thrown from, and run over, by a hand-car.

One of the local physicians, a conscientious and capable surgeon, was at once called, and discovered that the muscles of the injured man near to and parallel with the spinal column were badly lacerated, and that some of the bones of the column were fractured. The doctor promptly gave the attention and services necessary for the relief of his patient, but realizing that under the most favorable conditions an arduous task had fallen to his lot, asked the railway agent to wire the superintendent for the special authority required by the company in such cases.

The message was sent, but in place of instructions for the doctor to continue in charge, an imperative order was given the agent to send the man on first train to the Houston Infirmary.

I have no information as to who gave the order of removal, but as it was obeyed the presumption is legitimate that it came from some one in authority. The attending surgeon was not even notified of the intention to remove his patient, and, of course, had no opportunity to support the broken back by splints or plaster dressing, *nor to protest in the name of a noble profession* against such barbarous atrocity.

As might have been anticipated by anyone who had seen the wounded man, long before the train that bore him reached the destined point, death in mercy came to his relief.

There is no doubt in my mind, but that the official who gave the order of removal, was ignorant of the man's physical condition, and honestly believed that he was doing the best thing for him that could be done, but ignorance offers no excuse when positive information is obtainable.

*It was his duty to know the extent and character of the injury, before sending a telegram that had all the force, the consequences and solemn responsibilty of a death warrant.*

Medical men are about the only class who cannot, at any and all times, refuse to work when called upon. The lawyer, preacher, mechanic and laborer can, without offence, decline a service that dees not suit him ! But when crushed and mangled humanity calls for help, every impulse of a noble nature makes the call absolutely compulsory. At such times, compensation is an afterthought, and it is only regarded as a far off possibility, when those of us, unconnected with corporations, render service to the victims of railway accidents.

To prevent misunderstandings and delusive hopes upon that material point, the superintendent of the Houston and Texas Central, and probably other superintendents in the State, have issued special orders: *That no bills for services rendered to those injured by railroad accidents will be paid, unless explicit authority be given in each case to the person rendering the service*, or words to that effect. There is a steel-clad ring, a miserable selfishness, and a cold-blooded inhumanity about such orders that must be reflected upon, to be properly appreciated. It means, that any one, when hurt by their trains, must be patient in suffering until the circumstances of the injury are cautiously looked into, and then, if these high officials think the party injured worthy of consideration, they will, in their own way, give the needful help; if not worthy of consideration, if a poor tramp, or some moneyless unknown, *then the burden of caring for him must fall upon other shoutders.*

An accident occurred in this city, a few weeks ago, that enabled me to comprehend, as above indicated, *that special order!* and the subsequent history of the case suggested the writing of this article.

On the morning of August 19, one Peblo Mora was run over by an engine of the Houston and Texas Central Railway Company near the union depot.

Peblo Mora was an old Mexican, nearly blind, without wife or children, or any one who could sue for damages. He was struck as he crossed the track on Colorado street, by the tender of the engine. The engineer was backing, and could not, or did not see the old man, who was on a street crossing, a place where he not only had a right to be, but a place where some one at all times might be expected to be.

It was clearly the duty of the road management to have had a guard or guards at that time and place, to walk in front of advancing, and particularly in front of backing trains, to prevent such accidents; and in the absence of such customary precautions, the accident was the immediate result of wilful neglect. The engineer and local agents seemed to regret the casualty, and would have gladly rendered assistance, but they had no authority to do so, and the old man, with crushed and bleeding feet, was left to the mercy of those who had no connection with the injured, or with those inflicting the injury. A chance acquaintance had the wounded man removed to his cabin near by, and physicians of this place amputated the right leg, and performed Chopart's operation on the left foot. Notwithstanding the careful attention given, his death occurred on the third day after the accident. The city authorities had his remains decently interred—and the life, the history and the tragic death of Peblo Mora will soon pass from the memory of men! But humble and friendless though he was, if there is, as we are told, a faultless and incorruptible Tribunal beyond the grave, some one will there be arraigned for his cruel and inexcusable homicide.

A few days after the occurrences above narrated, I addressed to a prominent official in the medical department of the Central, an inquiry as to the steps to be taken in order to get some compensation for the money actually expended, and for the surgical services rendered by the doctors participating in the operation; and, as was expected, I received the prompt and laconic answer, that the General Superintendent of the H. & T. C. Railway Co. says, "that the company is not responsible for the injury done the Mexican, Peblo Mora, nor for the bills of his medical attendants." Even the burial expenses were repudiated, and the public money of the city was used to find a last resting place for the unfortunate man.

Had the injury occurred in a great wreck, where the eyes of the nation watched every painful incident connected with it, where the newspaper men were ready to turn their guns upon those who failed to meet the demands of the hour, where visions of damage suits appeared in the distance, we would have read, the day after the accident, how promptly surgeons had been summoned to care for even this stranger in a foreign land.

If a horse, or a cow, had been so killed on the streets of Austin, our city authorities could have compelled its removal by the railway officials. As it was only a friendless man, the corporation was not disturbed by the trifling episode.

If a private citizen had driven his wagon or carriage over that old Mexican, and had left him, with broken limbs, to die in the street, and had not at the time of injury, nor afterward, manifested the least interest in his fate; had refused to contribute one cent towards his comfort while living, or to the burial expenses when dead, what would this community, or any other civilized community, have thought and said about it? Every tongue would have pronounced the perpetrator a miserable brute! And yet, a corporation worth millions of dollars can fill out all those conditions without incurring either pecuniary loss or public condemnation.

If it is right to permit these giant corporations to thus impose upon benevolent people and upon city and county authorities, there is no need for statutory enactments; if it is not right, our law-makers should breathe into their inanimate corporate bodies legal souls that would make them respond, as mortals do, to cries of pity and distress.

The medical men of Texas necessarily see more of this railway injustice than others do, and upon them must devolve the duty of making a brave effort to correct it. We can urge upon our representatives, our senators, and upon our chief executive, the necessity of a law, compelling railway corporations *to promptly make comfortable provision for those injured by accidents, and to make said corporations pay all reasonable expenses that legitimately follow such injuries.* Some few roads in this State, voluntarily perform, to a humane extent at least, the very obligations herein suggested. By such a law, corporations disposed to do right would not be wronged, and corporations disposed to do wrong could be made to do right.

It could in no light be regarded as class legislation, nor a communistic infringement upon the rights of the rich; but a shield of protection that all governments should extend to their helpless and unfortunate subjects.

